# Text mining-based measurement of precision of polysomnographic reports as basis for intervention

**DOI:** 10.1186/s13326-022-00259-3

**Published:** 2022-01-31

**Authors:** Florent Baty, Jemima Hegermann, Tiziana Locatelli, Claudio Rüegg, Christian Gysin, Frank Rassouli, Martin Brutsche

**Affiliations:** 1 Lung Center, Cantonal Hospital St. Gallen, Rorschacherstrasse 95, St. Gallen, 9007 Switzerland; 2 Division of General Internal Medicine, Cantonal Hospital St. Gallen, Rorschacherstrasse 95, St. Gallen, 9007 Switzerland

**Keywords:** Text mining, Electronic medical reports, Polysomnography

## Abstract

**Background:**

Text mining can be applied to automate knowledge extraction from unstructured data included in medical reports and generate quality indicators applicable for medical documentation. The primary objective of this study was to apply text mining methodology for the analysis of polysomnographic medical reports in order to quantify sources of variation – here the diagnostic precision vs. the inter-rater variability – in the work-up of sleep-disordered breathing. The secondary objective was to assess the impact of a text block standardization on the diagnostic precision of polysomnography reports in an independent test set.

**Results:**

Polysomnography reports of 243 laboratory-based overnight sleep investigations scored by 9 trained sleep specialists of the Sleep Center St. Gallen were analyzed using a text-mining methodology. Patterns in the usage of discriminating terms allowed for the characterization of type and severity of disease and inter-rater homogeneity. The variation introduced by the inter-rater (technician/physician) heterogeneity was found to be twice as high compared to the variation introduced by effective diagnostic information. A simple text block standardization could significantly reduce the inter-rater variability by 44%, enhance the predictive value and ultimately improve the diagnostic accuracy of polysomnography reports.

**Conclusions:**

Text mining was successfully used to assess and optimize the quality, as well as the precision and homogeneity of medical reporting of diagnostic procedures – here exemplified with sleep studies. Text mining methodology could lay the ground for objective and systematic qualitative assessment of medical reports.

**Supplementary Information:**

The online version contains supplementary material available at (10.1186/s13326-022-00259-3).

## Background

Electronic medical reports constitute an important source of information for large scale healthcare quality studies [1, 2]. These reports generally include both structured/coded and unstructured/free-text information. Coded data can be easily summarized, whereas it is more challenging to extract pertinent information from free text. Narrative medical reports do not use standardized terminology and often contribute insufficient information for statistical processing and medical decision making [3]. The high diversity of terminology of unstructured medical reports leads to difficult extraction of information through computer processing and much information may be lost. Standardized terminology can help healthcare providers to obtain complete information and can improve healthcare quality [3]. Standardization methods can effectively increase data quality and reduce medical errors [4].

Several attempts have been proposed to unify and control the medical vocabulary. Medical Subject Headings (MeSH), used by MEDLINE, is employed for the purpose of indexing journal articles in life science. International Classification of Disease (ICD) is another classification system of diseases. Methodological approaches have been described in the literature aiming to facilitate the exploration of narrative texts included in electronic health records (EHR) (see e.g. [5]). These works typically stress the difficulty to extract insightful information from EHR due to the complexity of the information (codified text, use of jargon jerky terminology, etc.).

Text mining (TM) refers to the process of deriving meaningful insights from textual sources. This process encompasses several analytical challenges including retrieving, annotating, exploring and interpreting valuable information from text corpora. TM can be applied to automate knowledge extraction from unstructured data included in medical reports and generate quality indicators applicable for medical documentation [6–9]. Free text description of complex diseases reported in health records can be subject to various sources of variation. It is of interest to keep the text as accurate and standardized as possible in order to minimize errors, miscoding and loss of information susceptible to have a negative impact on patient management.

Sleep apnea (SA) is a prevalent sleep disorder characterized by a reduction or cessation of airflow to the lungs caused by obstructive or central events. SA is diagnosed by polysomnography (PSG) based on the number of apnea-hypopnea events per hour of sleep. PSG is technically complex. This procedure generates elaborated reports whose interpretation requires the expertise of sleep technicians under the supervision of trained physicians.

Applications of TM in the field of sleep disorders exist but are scarce. For example, TM methodology was applied for the determination of trendy sleep disorder terminologies in recent sleep-related journal articles [10]. Moreover, sleep domain ontology proposed on the NCBO BioPortal provides a set of controlled vocabulary (English language) with specific application on sleep medicine [11].

The aim of the current study was to apply TM to PSG medical reports for quality purposes. More specifically, the aim was to assess the inter-rater variability in the diagnostic evaluation of sleep-disordered breathing by quantifying the part of variation associated with objective patient’s diagnosis (type of disease, disease severity) and comparing it with the part of variation explained by the subjective rater’s interpretation. In a second step, we sought to reduce the inter-rater variability in an independent test set by text standardization.

## Results

### Text mining of pSG reports

**Fig. 1:**
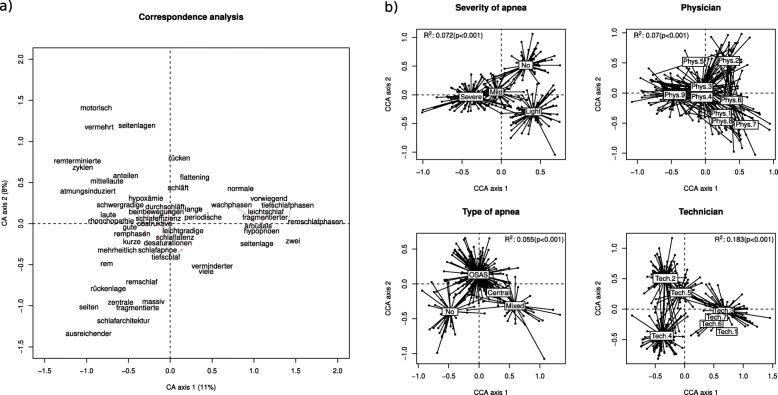
Correspondence analysis (CA) of the term-document matrix. The left panel (**a**) displays the term scores on the first 2 CA axes. The right panel (**b**) shows a ‘spider’ diagram connecting each level of the explanatory variables (apnea severity, physicians, type of apnea and technicians) to its group centroid on both main axes of the canonical correspondence analysis

Overall, 695 unique terms were extracted from the corpus of PSG medical reports among which 52 keywords were retained based on their usage frequency (all terms whose sparsity was greater than 90% were removed). The list of discriminating terms is provided in the Additional file 1 (Additional Table 1). A term-document matrix (243 documents × 52 terms) was created and analyzed using CA (data and source codes are provided in the Additional files 2 and 3). Figure 1a displays the term usage ordinated by CA. The first 2 CA axes summarized 11% and 8% of the overall variation, respectively. The percentage of variance explained by the disease characteristics (diagnosis and severity) was 6% and 7%, respectively. On the other hand, the percentage of variance explained by the raters (technicians and physicians) was 18% and 7%, respectively (Fig. 1b). Noteworthily, clustering among technicians (1, 3, 6 and 7) and among physicians (1, 6, 7 and 8) could be observed, showing some similarities in the semantic of polysomnographic reports among technicians/physicians.

### Effect of text block standardization

**Fig. 2:**
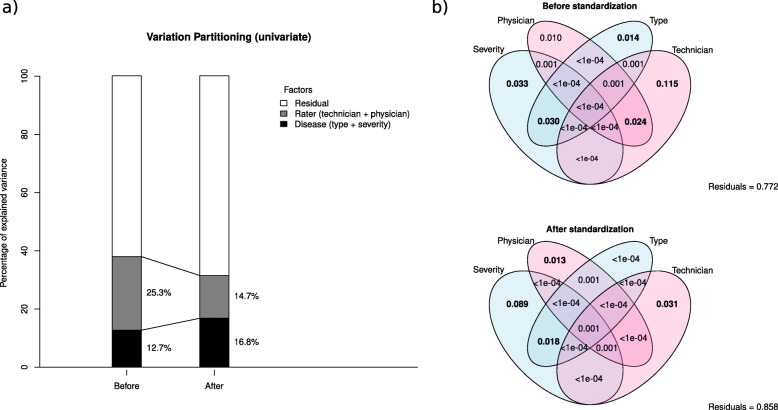
Variation partitioning before and after text block standardization of the polysomnography reports. The left panel (**a**) shows the effect of text block standardization on the percentage of variance explained by the raters and disease characteristics. The right panel (**b**) provides details on the fractions of variation between the different explanatory variables before (upper plot) and after (lower plot) text block standardization using Venn diagram representations

After text block standardization, the total variance measured by the total inertia of the correspondence analysis, decreased from 2.73 down to 1.13. The percentage of variance explained by the raters (technicians / physicians) dropped from 25% to 15%, whereas the percentage of variance explained by the disease characteristics (type of apnea / disease severity) increased from 13% to 17% (Fig. 2a). The fractions of variation between the explanatory variables are shown in Fig. 2b. Before standardization, the combined percentage of the explanatory variables associated with the objective patient’s diagnosis (type of apnea / severity) represented 8% of the overall variation whereas 14% of the total variation was associated with subjective interpretation of sleep technicians and physicians. After standardization, the percentage of explained variance associated with the disease increased to 11%, whereas the percentage of explained variance associated with rater decreased down to 4%. The ratio of disease to rater explained variance favorably increased from 0.5 to 2.75.

The predictive accuracy of the final SA diagnosis was assessed using a linear support vector machine classifier with a repeated 10-fold cross-validation. Patients were classified in the following 6 diagnostic categories: obstructive sleep apnea (OSAS) light (*n*=13), OSAS mild (*n*=18), OSAS severe (*n*=45), central SA (*n*=4), mixed SA (*n*=4) and undetected SA (*n*=16). Overall, an accuracy of 88% (95% CI: 83 to 91) was obtained when using the standardized text compared with 86% (95% CI: 83 to 88) without standardization. The confusion matrix of the cross-validation procedure is provided in Table 1. The prediction accuracy was particularly high with regard to the three subclasses of obstructive sleep apnea (light, mild and severe) and for the prediction of cases without detected apnea events. On the other hand, SA patients including central or mixed events were more difficult to predict. In some cases, central SA was misclassified as severe/light OSAS or mixed SA, whereas some mixed SA were misclassified as severe OSAS. These misclassifications can be partially explained by the low number of patients diagnosed with a central/mixed SA in the current study. The restriction in the terms selection used in the standardized reports numerically improved the diagnostic accuracy of the final SA diagnosis.

**Table 1:** Cross-validated confusion matrix summarizing the predictive value of the standardization procedure

	Reference					
Prediction	Central SA	Mixed SA	Undetected	OSAS/light	OSAS/mild	OSAS/severe
Central SA	0.00	0.00	0.00	0.00	0.00	1.33
Mixed SA	0.33	1.67	0.00	0.00	0.00	0.00
Undetected	0.00	0.00	14.33	2.33	0.67	0.00
OSAS/light	1.00	0.00	0.33	10.67	0.00	0.00
OSAS/mild	0.00	0.00	0.33	0.00	17.33	0.00
OSAS/severe	2.67	2.33	1.00	0.00	0.00	43.67
Pred. accuracy (%)	0.0	41.7	89.6	82.1	96.3	97.0

The table entries report the percentual average cell counts across resamples following a 10-fold cross-validation with 3 repetitions. The bottom line provides the class-wide prediction accuracy

## Discussion

Electronic health reports contain information about patient’s condition, which can be retrieved in an automatic manner [12]. However, unstructured text included in medical reports is often hampered by a series of pitfalls related among others to the raters’ narrative style [13], the ambiguity or the redundancy of the reported information [14], the customization of the texts and the clinical experience of the rater.

This inter-rater language heterogeneity is a potential source of confusion when extracting objective medical information from a health report. It is in the interest of quality assurance to maximize the diagnostic precision, *i.e.* the proportion of objective (disease / severity) over subjective (rater) information content included in health reports. TM can lay the ground for the evaluation of measures to efficiently standardize the information present in medical reports (*e.g.* using text blocks combined with the unified medical language system [15]), and minimize the risk of imprecision.

With TM methodology it is possible to quantify the importance of several sources of variation present in medical reports. In the current study, the variation introduced by inter-rater (technician/physician) heterogeneity was found to be twice higher compared to the variation introduced by effective diagnostic information. In order to improve the consistency of the PSG medical reports, we found that further standardization of the reporting in the form of a semi-structured documentation could improve the homogeneity and objectivity of generated reports, with a high predictive value, while maintaining the possibility of adding free text comments when needed.

There are several limitations to the current study. Discriminating terms were extracted from the corpus of documents based on automated procedures and did not include further meticulous manual inspections. Although this basic methodological approach was deemed sufficient within the scope of the current study, future developments could include more advanced data curation such as stemming and other refined text transformations. Future works on structured medical reports could also benefit from the use of controlled medical vocabulary.

## Conclusion

The analysis of electronic health reports with text mining techniques combined with correspondence analysis and variance partitioning provides a unique and powerful way to assess and optimize the quality of medical reporting. To the best of our knowledge, this is the first time that such an approach has been applied in the field of sleep medicine. Generalization of strategies of text analytics in healthcare should be encouraged as they trigger quality improvements in most health systems with a direct benefit for clinicians and patients.

## Methods

### Polysomnography reports

In a retrospective quality survey, 243 PSG medical reports were retrieved from the Sleep Center of the Cantonal Hospital St. Gallen. These reports were taken from consecutive patients with suspicion of SA referred for a whole-night PSG. All patients were included in a prior study investigating the clinical validity of a novel wearable electrocardiogram (ECG) device [16–18]. The study was performed in accordance with the Declaration of Helsinki, following the principles of Good Clinical Practice. The study was approved by the local institutional review board (EKSG 15/140) and patients gave written informed consent to participate. Patients data were analyzed in a fully anonymized manner.

Altogether, the PSG medical reports were assessed by 7 sleep technicians and validated by 9 sleep physicians. Diagnoses included obstructive, central and mixed sleep apnea with various levels of severity. Data from PSG records are evaluated by sleep technicians based on information presented in the form of tables and graphics. Technicians typically provide a provisional interpretation of the sleep record, highlighting the main features and characteristics. This initial interpretation is thereafter validated by a pulmonologist who adapts and corrects the report if necessary. A snapshot of an example of PSG report is provided in the Additional file 4 (Snapshot of a PSG medical report). The narrative interpretation is highlighted in the bottom inset.

### Text block standardization

A standardization of the PSG reports was implemented using predefined blocks of text sequentially assessing sleep features in a systematic manner. The resulting standardized approach – thereafter called *text block standardization* – increases the uniformity of the diagnostic information contained in these reports. This standardization automates the generation of PSG reports with a systematic sequential description of the following items: sleep latency (normal, shortened, lengthened), sleep efficiency (normal, reduced), sleep architecture (fragmented, shortened, with lack of rapid eye movement [REM] phase), sleep stages and position in which the patient slept (lateral position, on the back, on the abdomen). Thereafter, it is described whether the patient had an obstructive, mixed or central sleep apnea, together with indications on the sleep apnea severity (mild, moderate, severe) and whether sleep apnea was associated with the patient’s position and/or REM phase. Furthermore, the following items are highlighted: oxygen saturation, hypoxemia and hypercapnia, presence of snoring, arousal index and presence of periodic movements of the lower limbs. The specialized pulmonologist finally checks (and possibly adapt/correct) the automatically generated report. For the purpose of the current analysis, one hundred consecutive reports from independent patients were extracted.

### Statistical approaches

#### Text mining approach

The narrative section of PSG electronic reports was extracted and analyzed using TM. TM summarizes the usage of key terms throughout a corpus of textual documents by generating a term-document matrix. More specifically, TM requires several pre-processing steps of data cleansing [19]. The TM procedure used in the current study follows the guidelines provided in the vignette of the R package tm [20]. The procedure includes the elimination of extra white spaces, stop words, common words in the German language, punctuation, numbers, sparse terms and transformation to lower case terms. The filtered terms were cross-tabulated in a term-document matrix. The term-document matrix tend to be very large and, as suggested in the introductory guidelines of the R-package tm, a step consisting in removing sparse terms occurring only in few documents can be employed to reduce the matrix without losing significant relations inherent to the matrix.

#### (Constrained-)correspondence analysis and variation partitioning

The term-document matrix was analyzed using correspondence analysis (CA), a multivariate dimension reduction method appropriate for the analysis of contingency tables. Theoretical aspects underlying CA can be summarized by defining the following:

- **X** the *n*×*m* term-document matrix (*n* documents, *m* terms)
- **P**=**X**/**N** the data matrix divided by its grand total ($\mathbf {N} = \sum _{i=1}^{n} \sum _{j=1}^{m} x_{ij}$, the sum of all elements in **X**)
- **r** the *n*-dim vector of row sums of **P** (row weights)
- **c** the *m*-dim vector of row sums of **P** (column weights)
- **D**_*r*_ the *n*×*n* diagonal matrix of row sums
- **D**_*c*_ the *m*×*m* diagonal matrix of column sums

In CA, the main table of interest (term-document matrix) is converted into a *χ*^2^ distance matrix after performing the following transformation:

$$\mathbf{Z} = \mathbf{D}_{r}^{-1/2} (\mathbf{P} - \mathbf{rc}^{\top}) \mathbf{D}_{c}^{-1/2} $$

CA consists in the singular value decomposition of **Z**:

$$\mathbf{Z} = \mathbf{U}\mathbf{\Lambda}\mathbf{V}^{\top} $$

with **Λ** the *k*×*k* (*k*=*rank*(**Z**)) diagonal matrix of singular values associated with **Z** with *λ*_1_≥⋯≥*λ*_*k*_>0,**U** the *n*×*k* matrix of left singular vectors and **V** the *m*×*k* matrix of right singular vectors. The total inertia of the contingency table is given by the sum of the squared singular values ($I = \sum _{i=1}^{p} \lambda _{i}^{2}$, with *p* the smaller dimension of **X**).

The contingency table was partitioned with respect to explanatory variables using variation partitioning techniques [21]. The following four explanatory variables were considered: type of apnea, apnea severity, physician, technician. The partitioning was based on constrained correspondence analysis (CCA), a supervised counterpart of CA (*e.g.*, [22]). In CCA, linear constraints are applied observation-wise. Each categorical explanatory variable is used to define row blocks. If we define **M** the *n*×*g* matrix of dummy variables defining *g* blocks among observations, the observation-wise constraint is given by the projection operator:

$$\mathbf{O}_{r} = \mathbf{M} (\mathbf{M}^{\top} \mathbf{D}_{r} \mathbf{M})^{-1} \mathbf{M}^{\top} \mathbf{D}_{r} $$

The projection on **O**_*r*_ computes the means per block of observations for each variable. CCA consists in performing the following singular value decomposition:

$$\mathbf{Z}^{*} = \mathbf{D}_{r}^{-1/2} \mathbf{O_{r}} (\mathbf{P} - \mathbf{rc}^{\top}) \mathbf{D}_{c}^{-1/2}=\mathbf{U}^{*}\mathbf{\Lambda}^{*}\mathbf{V}^{*\top} $$

with **Λ**^∗^ the *k*^∗^×*k*^∗^ (*k*^∗^=*rank*(**Z**^∗^)) diagonal matrix of singular values associated with **Z**^∗^ with $\lambda _{1}^{*} \ge \cdots \ge \lambda _{k}^{*} > 0, \mathbf {U}^{*}$ the *n*×*k*^∗^ matrix of left singular vectors and **V** the *m*×*k*^∗^ matrix of right singular vectors.

The percentage of explained variance associated with a specific explanatory variable is given by the ratio of the total inertia of constrained over unconstrained CA. In a first step, the total inertia of CA was partitioned according to each explanatory variable using univariate analyses and the reported percentage of explained variance corresponded to the unadjusted *R*-squared, *i.e.* the fraction of variance explained by each individual explanatory variable independently of the other variables. In a second step, adjusted *R*-squared were calculated where the joint effect among variables was taken into account. For each explanatory variable, the percentage of explained variance and its significance was assessed using permutation tests. The inter-rater variability was defined by the percentage of explained variance associated with both physicians and technicians.

#### Predictive accuracy of the final diagnosis

The predictive value of the text standardization was assessed using a linear support vector machine (SVM) classifier and the prediction accuracy of the classifier was estimated using repeated 10-fold cross-validation. In 10-fold cross-validation, the original sample is randomly partitioned into 10 equal size subsamples. Of the 10 subsamples, 1 single subsample is retained as test data and the remaining 9 subsamples are used as training data. The process is repeated 10 times, each subsample being used exactly once as validation test data. All observations are used both for training and validation. Furthermore, the cross-validation procedure was repeated 3 times. The SVM-classifier and its cross-validation was implemented using the function *train* of the R package caret using the following control parameters: resampling method was set to *“repeatedcv”*, number of folds was set to *10* and number of repetitions of *k*-fold was set to *3*. The following diagnostic classes were considered: OSAS severe, OSAS mild, OSAS light, central SA, mixed SA, undetected SA. The class distribution and detailed class-wise performance was provided.

#### Statistical software implementations

Source codes can be provided upon request to the corresponding authors. All analyses were done using the R statistical software (v. 4.0.3) including the following extension packages: tm [23], ade4 [24], vegan [25] and caret [26]. CA was performed using the function *dudi.coa* implemented in ade4, and CCA using the function *cca* implemented in vegan. Variation partitioning was performed using the function *varipart* implemented in ade4. Source codes can be provided upon request to the corresponding authors.

## Supplementary Information

**Additional file 1** Additional Table 1: Discriminating terms used in the original analysis.

**Additional file 2** Additional Data 1: Term-document matrix.

**Additional file 3** Additional Source Code 1: Supportive R source code needed to perform the correspondence analysis.

**Additional file 4** Snapshot of a PSG medical report: Example of PSG medical report including a narrative description of the whole-night investigation.

## Data Availability

The data sets generated during and/or analyzed during the current study are not publicly available due to data privacy issue related to the nature of the analyzed patient information but are available from the corresponding author on reasonable request.

## Abbreviations

CA: Correspondence analysis
CCA: Constrained correspondence analysis
ECG: Electrocardiogram
EHR: Electronic health records
EKSG: Ethikkommission St. Gallen
MeSH: Medical subject headings
NCBO: National Center for Biomedical Ontology
OSAS: Obstructive sleep apnea
PSG: Polysomnography
REM: Rapid eye movement
SA: Sleep apnea
TM: Text mining

## References

[1] Ford E, Carroll JA, Smith HE, Scott D, Cassell JA (2016). Extracting information from the text of electronic medical records to improve case detection: a systematic review. J Am Med Inform Assoc.

[2] Miotto R, Wang F, Wang S, Jiang X, Dudley JT (2018). Deep learning for healthcare: review, opportunities and challenges. Brief Bioinform.

[3] Přečková P, Zvárová J, Zvára K (2012). Measuring diversity in medical reports based on categorized attributes and international classification systems. BMC Med Inform Decis Mak.

[4] Kuru K, Girgin S, Arda K, Bozlar U (2013). A novel report generation approach for medical applications: the SISDS methodology and its applications. Int J Med Inform.

[5] Quimbaya AP, Múnera AS, Rivera RAG, Rodríguez JCD, Velandia OMM, Peña AAG, Labbé C (2016). Named entity recognition over electronic health records through a combined dictionary-based approach. Procedia Comput Sci.

[6] Cohen AM, Hersh WR (2005). A survey of current work in biomedical text mining. Brief Bioinform.

[7] Raja U, Mitchell T, Day T, Hardin JM (2008). Text mining in healthcare. Applications and opportunities. J Healthc Inf Manag.

[8] Zhu F, Patumcharoenpol P, Zhang C, Yang Y, Chan J, Meechai A, Vongsangnak W, Shen B (2013). Biomedical text mining and its applications in cancer research. J Biomed Inform.

[9] Pereira L, Rijo R, Silva C, Martinho R (2015). Text mining applied to electronic medical records: A literature review. Int J E-Health Med Commun (IJEHMC).

[10] Lam C, Lai FC, Wang CH, Lai MH, Hsu N, Chung MH (2016). Text Mining of Journal Articles for Sleep Disorder Terminologies. PLoS ONE.

[11] Sleep Domain Ontology. 2010. https://bioportal.bioontology.org/ontologies/SDO. Accessed 19 May 2021.

[12] Delespierre T, Denormandie P, Bar-Hen A, Josseran L (2017). Empirical advances with text mining of electronic health records. BMC Med Inform Decis Mak.

[13] Kovacevic A, Dehghan A, Filannino M, Keane JA, Nenadic G (2013). Combining rules and machine learning for extraction of temporal expressions and events from clinical narratives. J Am Med Inform Assoc.

[14] Cohen R, Elhadad M, Elhadad N (2013). Redundancy in electronic health record corpora: analysis, impact on text mining performance and mitigation strategies. BMC Bioinformatics.

[15] Bodenreider O (2004). The Unified Medical Language System (UMLS): integrating biomedical terminology. Nucleic Acids Res.

[16] Baty F, Boesch M, Widmer S, Annaheim S, Fontana P, Camenzind M, Rossi RM, Schoch OD, Brutsche MH (2020). Classification of Sleep Apnea Severity by Electrocardiogram Monitoring Using a Novel Wearable Device. Sensors (Basel).

[17] Fontana P, Martins NRA, Camenzind M, Boesch M, Baty F, Schoch OD, Brutsche MH, Rossi RM, Annaheim S (2019). Applicability of a Textile ECG-Belt for Unattended Sleep Apnoea Monitoring in a Home Setting. Sensors (Basel).

[18] Fontana P, Martins NRA, Camenzind M, Rossi RM, Baty F, Boesch M, Schoch OD, Brutsche MH, Annaheim S (2019). Clinical Applicability of a Textile 1-Lead ECG Device for Overnight Monitoring. Sensors (Basel).

[19] Sun W, Cai Z, Li Y, Liu F, Fang S, Wang G (2018). Data Processing and Text Mining Technologies on Electronic Medical Records: A Review. J Healthc Eng.

[20] Feinerer I, Hornik K. tm: Text Mining Package. 2018. R package version 0.7-6. https://CRAN.R-project.org/package=tm. Accessed 19 Jan 2022.

[21] Peres-Neto PR, Legendre P, Dray S, Borcard D (2006). Variation partitioning of species data matrices: estimation and comparison of fractions. Ecology.

[22] Baty F, Ritz C, Charles S, Brutsche M, Flandrois J-P, Delignette-Muller M-L (2015). A toolbox for nonlinear regression in r: The package nlstools. J Stat Softw Artic.

[23] Feinerer I, Hornik K, Meyer D (2008). Text mining infrastructure in R. J Stat Softw.

[24] Dray S, Dufour A-B (2007). The ade4 package: Implementing the duality diagram for ecologists. J Stat Softw.

[25] Oksanen J, Blanchet FG, Friendly M, Kindt R, Legendre P, McGlinn D, Minchin PR, O’Hara RB, Simpson GL, Solymos P, Stevens MHH, Szoecs E, Wagner H. Vegan: Community Ecology Package. 2019. R package version 2.5-6. https://CRAN.R-project.org/package=vegan. Accessed 19 Jan 2022.

[26] Kuhn M. Caret: Classification and Regression Training. 2020. R package version 6.0-86. https://CRAN.R-project.org/package=caret. Accessed 19 Jan 2022.

