# Supplementary material for: Text mining-based measurement of precision of polysomnographic reports as basis for intervention
Source: J Biomed Semantics. 2022 Jan 31;13:5. doi: 10.1186/s13326-022-00259-3 (PMC8805265; doi:10.1186/s13326-022-00259-3)
Supplement: Supplementary file 1 — Additional file 1 Additional Table 1: Discriminating terms used in the original analysis. [file 13326_2022_259_MOESM1_ESM.pdf]

Text mining-based measurement of diagnostic precision of polysomnographic reports as basis for intervention
Additional File 1

F. Baty, J. Hegermann, T. Locatelli, C. Rüegg, C. Gysin, F. Rassouli, M. Brutsche

2021-05-25

Additional Tables

Table 1: Discriminating terms used in the original analysis.

Terms
anteilen
arousals
atmungsinduziert
ausreichender
beinbewegungen
desaturationen
durchschläft
flattening
fragmentierte
fragmentierter
gute
hypopnoen
hypoxämie
kurze
lange
laute
leichtgradige
leichtschlaf
massiv
mehrheitlich
mittellaute
motorisch
normale
obstruktive
periodische
rem
remphasen
remschlaf
remschlafphasen
remterminierte
rhonchopathie
rücken
rückenlage

Terms
schlafapnoe
schlafarchitektur
schlafeffizienz
schlaflatenz
schläft
schwergradige
seiten
seitenlage
seitenlagen
tiefschlaf
tiefschlafphasen
vermehrt
verminderter
viele
vorwiegend
wachphasen
zentrale
zwei
zyklen
