# Supplementary figures and images for: Text mining-based measurement of precision of polysomnographic reports as basis for intervention
Source: J Biomed Semantics. 2022 Jan 31;13:5. doi: 10.1186/s13326-022-00259-3 (PMC8805265; doi:10.1186/s13326-022-00259-3)

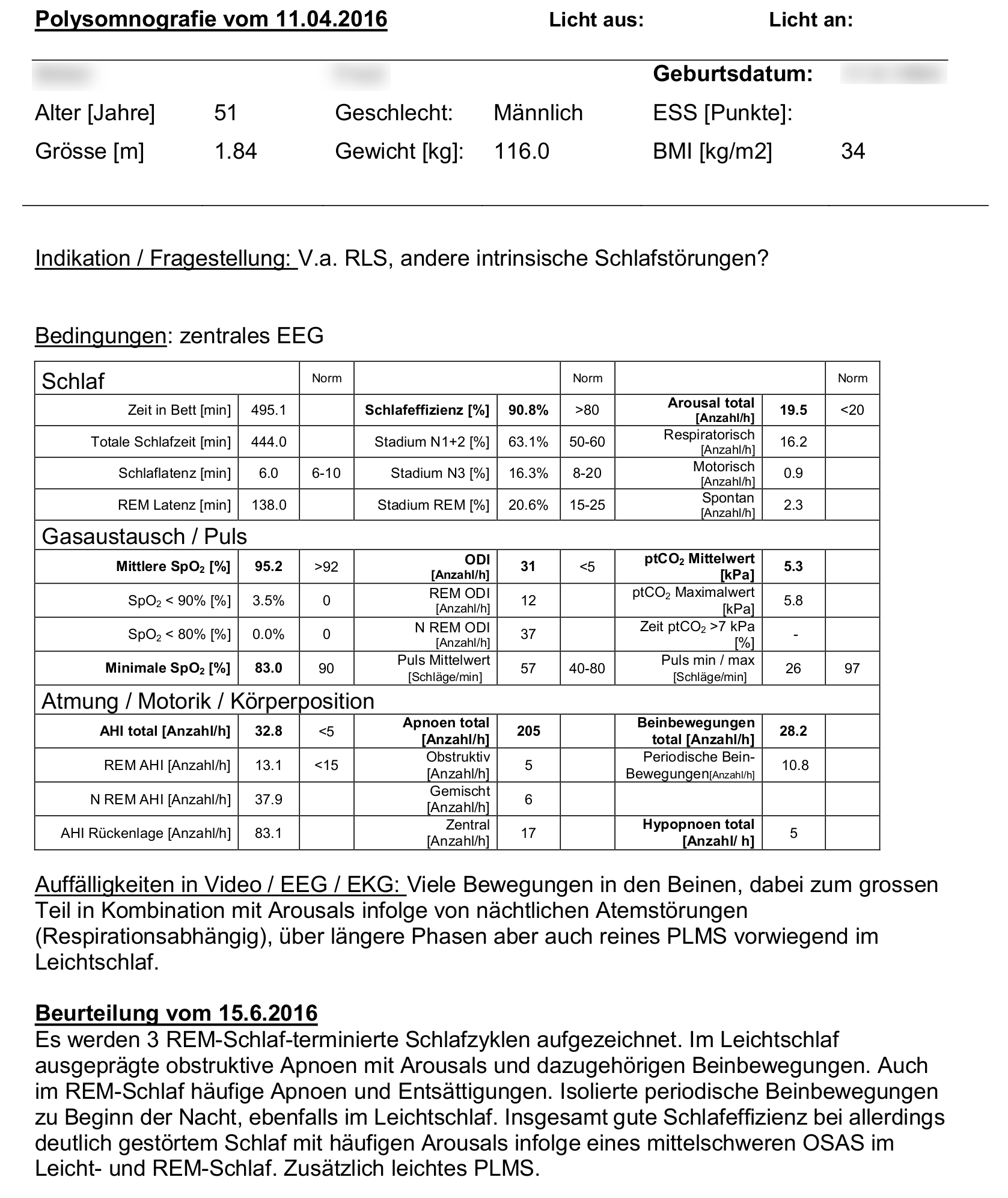

Supplement: Supplementary file 4 — Additional file 4 Snapshot of a PSG medical report: Example of PSG medical report including a narrative description of the whole-night investigation. [file 13326_2022_259_MOESM4_ESM.png]
